# Supplementary material for: Using Alphafold2 to Predict the Structure of the Gp5/M Dimer of Porcine Respiratory and Reproductive Syndrome Virus
Source: Int J Mol Sci. 2022 Oct 30;23(21):13209. doi: 10.3390/ijms232113209 (PMC9653971; doi:10.3390/ijms232113209)
Supplement: Supplementary file 1 [file ijms-23-13209-s001.zip › ijms-1916395-supplementary.pdf]

Figure S1

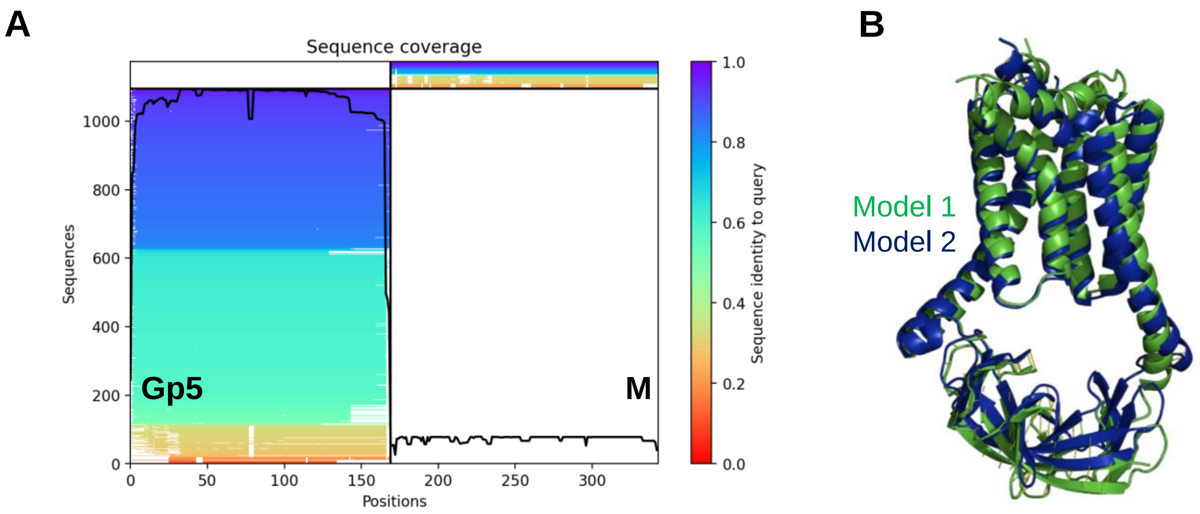

Figure S1: MSA for Gp5/M of VR 2332 and superposition of the best two models
A: Multiple sequence alignment (MSA) file for the prediction of the Gp5/M dimer of PRRSV-2 strain VR 2332. The number of sampled sequences on the y-axis is plotted against the amino acid position on the X-axis. The bar on the right indicates the relative sequence identity to the queried sequence, colour coded from red (low identity) to blue (high identity). The black line indicates the sequence coverage, which tends to be lower at the N- and C-terminus of both proteins because some genes were not completely sequenced.
B: The model 1 (green cartoon) of the Gp5/M from VR 2332 dimer is superimposed with the model ranked 2 (blue cartoon) using the align function of PyMol.

Figure S2

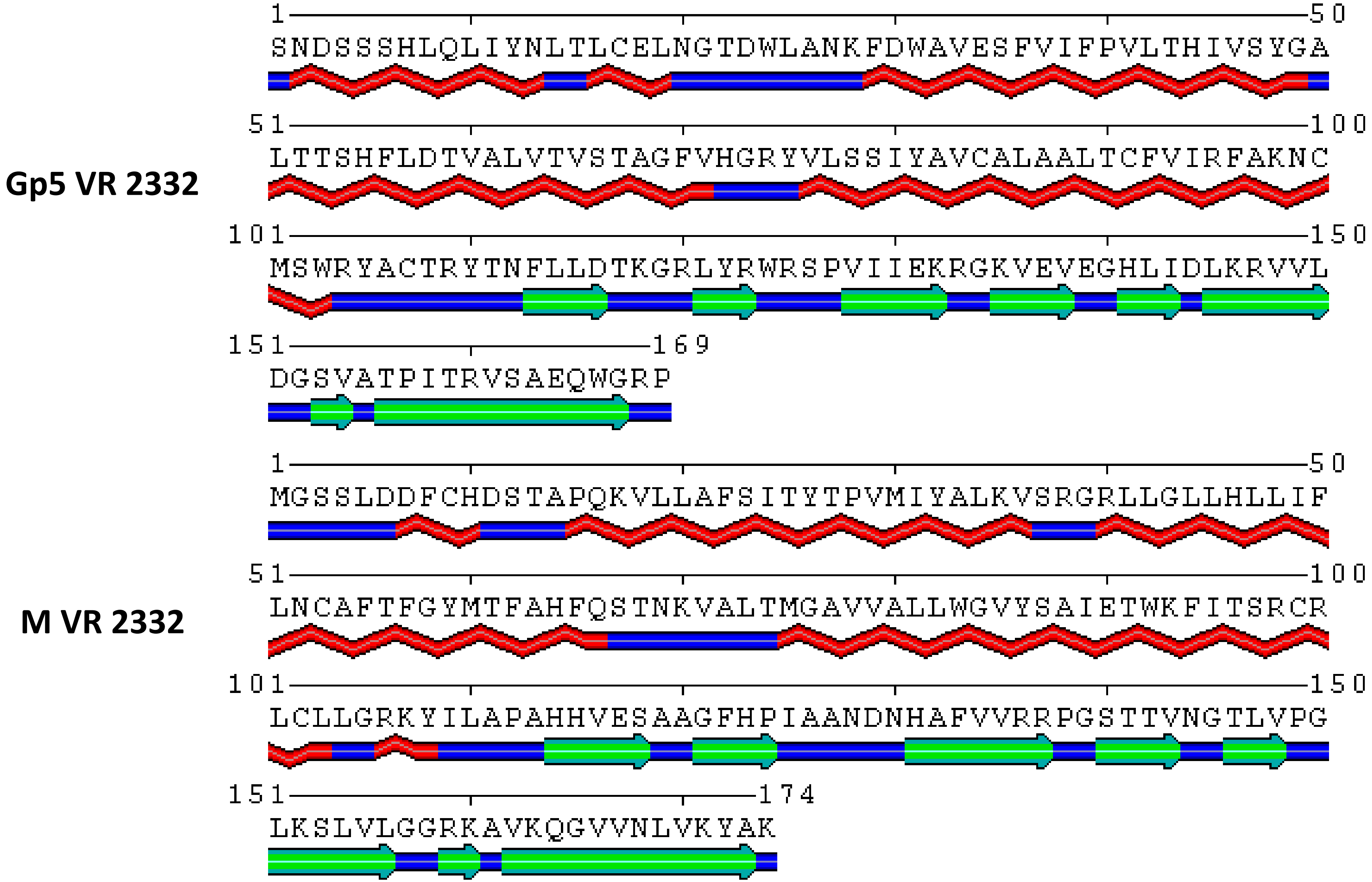

Figure S2: Assignment of the Gp5 and M sequence of VR 2332 to the structural elements
Assignment of the Gp5 and M sequence of VR 2332 to the structural elements predicted by model 1. Red: α-helices, green: β-strands, blue: no structure, loops.

Figure S3

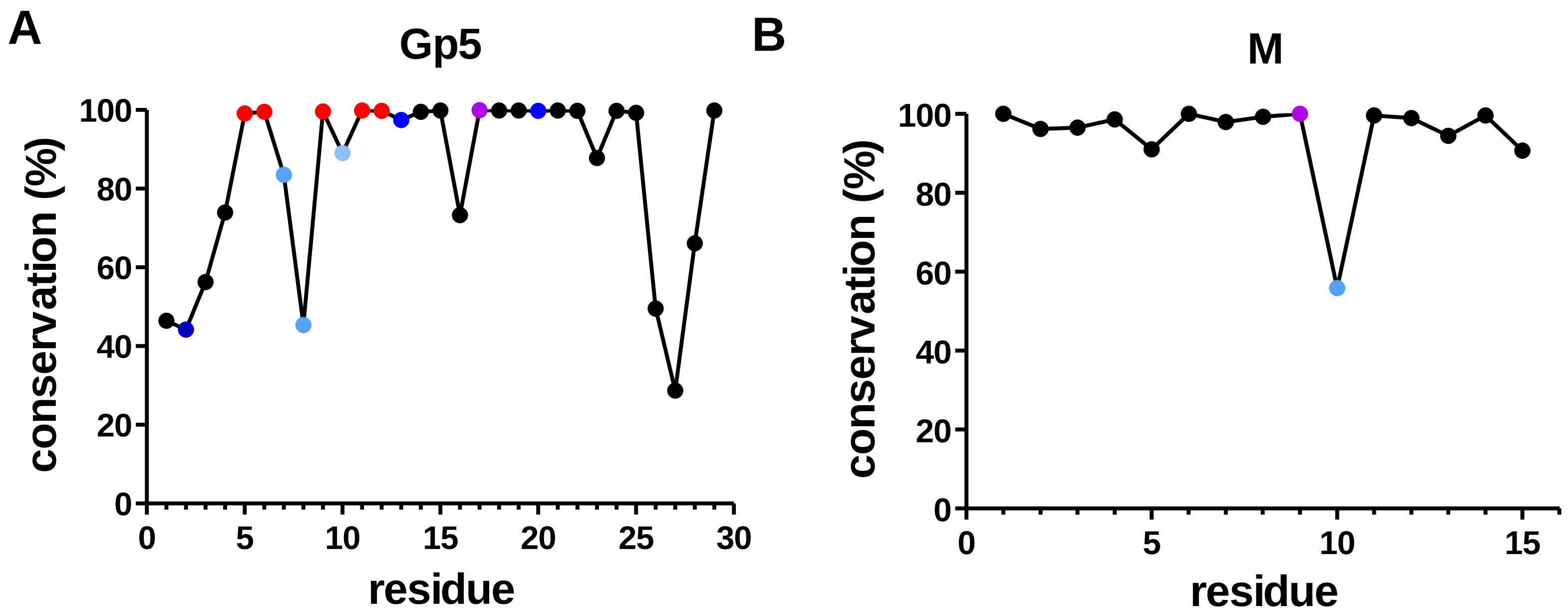

Figure S3: Conservation of amino acids in the ectodomain of Gp5 and M of PRRSV-2 strains.
The graphs show the percent conservation (y axis) of amino acids at each position (x axis) of a consensus sequence compiled from 7701 Gp5 (A) and 290 M (B) sequences of PRRSV-2 present in the database. Dark blue: N-glycosylation sites, magenta: cysteine residues. Red and cyan in Gp5: neutralizing epitope showing conserved (red) and variable (cyan) residues. Cyan: Residue 10 in M, where amino acid exchange leads to escape from broadly neutralizing antibodies.
See figure 3c and d for a web logo representation showing the amino acids present at each position.

Figure S4

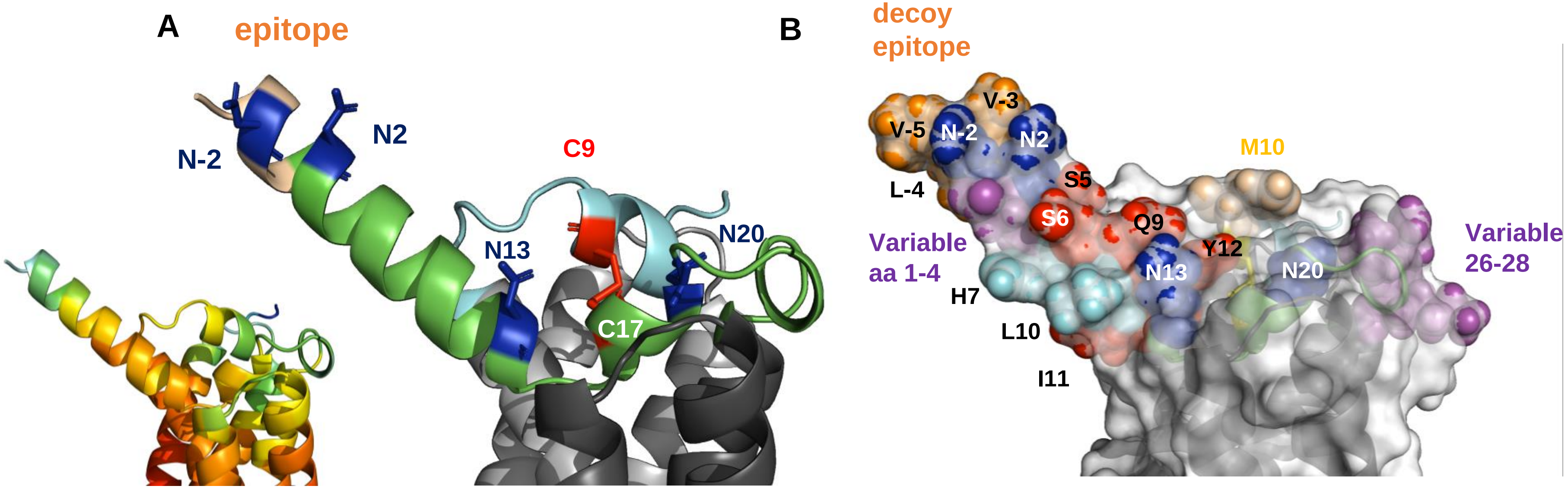

Figure S4: Structure of the ectodomain of Gp5/M of VR 2332 including the decoy epitope

A: Cartoon model. Hydrophilic residues in Gp5 are colored green and in M cyan. Hydrophobic membrane parts of Gp5 in dark grey and of M in light grey. N-glycosylated Asn are shown as blue sticks, the disulfide-bond between Gp5 and M as red sticks. Inset: quality of the model displayed in rainbow colors ranging from red (high confidence) to blue (low confidence).

B: Surface representation of the ectodomain. N-glycosylation sites are shown as blue spheres, between PRRSV-2 strains variable amino acids as magenta spheres. The neutralizing epitope (residues 5-12) is also highlighted, conserved residues as red spheres and between strains variable residues as cyan spheres. Residue 10 of M, which is also involved in antibody binding, is shown as a wheat sphere. The decoy-epitope is shown as orange spheres. Note that the numbering of residues corresponds to the structure excluding the decoy-epitope, e.g. V-5 is the first amino acid in this structure.

Figure S5

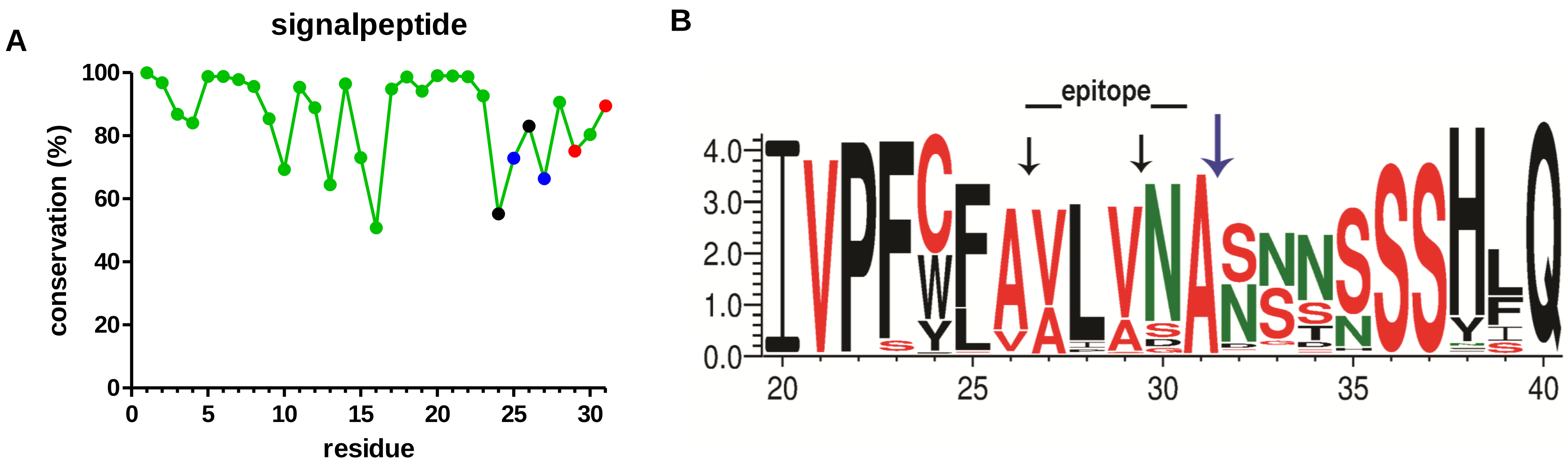

Figure S5: Conservation of amino acids in the signal peptide of Gp5 of PRRSV-2 strains
A: The graphs show the percent conservation (y axis) of amino acids at each position (x axis) of a consensus sequence compiled from 7701 GP5 sequences of PRRSV-2 present in the database. Red, blue and black dots: pairs of small amino acids that are in principle suitable as signal peptide cleavage site.
B: Web loge representation of amino acids 20 to 40 of full-length Gp5 sequences including the signal peptide. The blue, large arrow indicate the main cleavage site predicted by SignalP5 and the minor black arrows additional predicted cleavage sites. Small amino acids are shown in red and Asn residues as putative N-glycosylation sites are shown in green. The location of the decoy epitope is indicated by a black line.

Figure S6

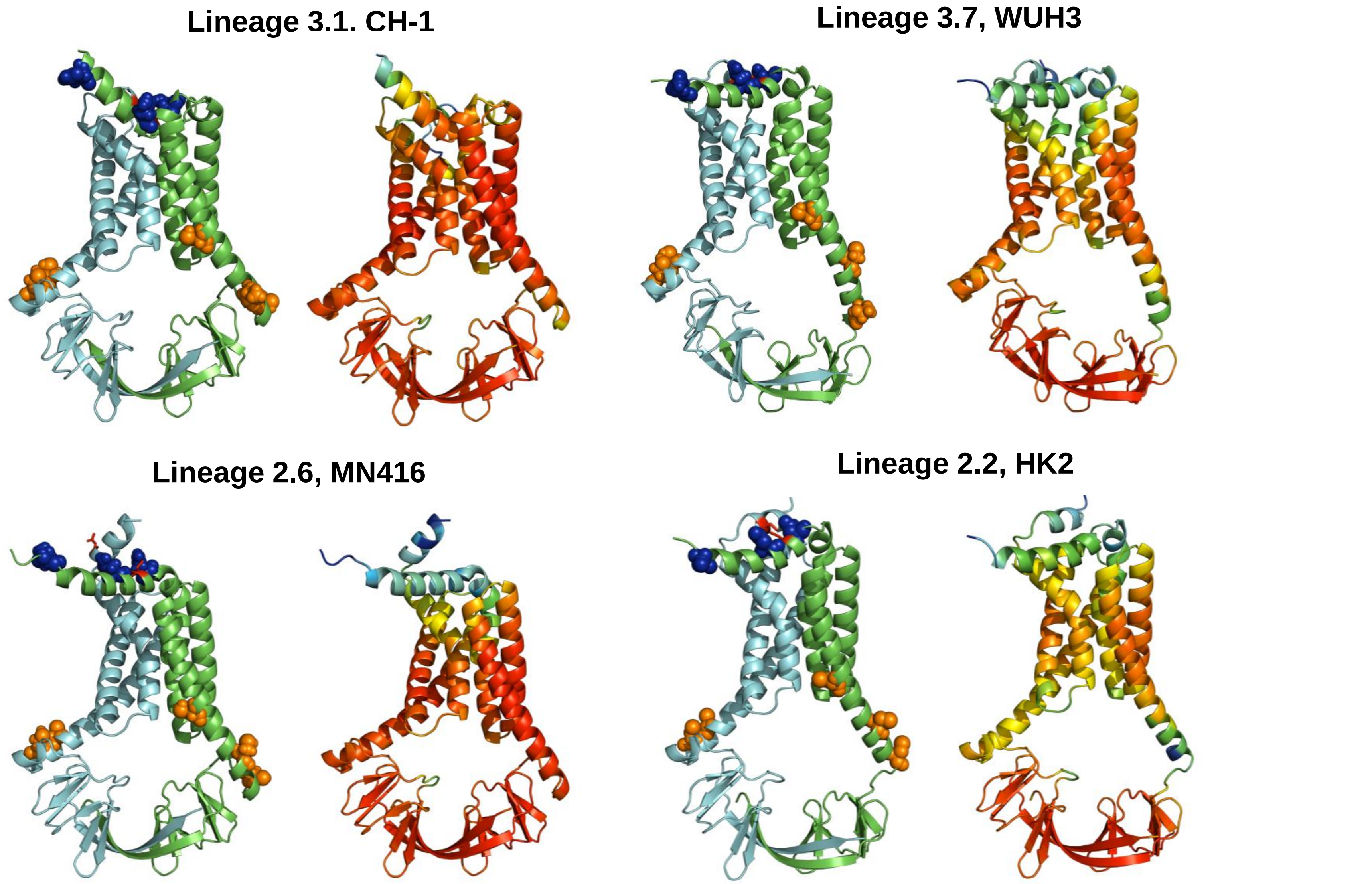

Figure S6: Structure of Gp5/M of representative members of four different lineages of PRRSV-2
Left part: Cartoon model. Gp5 is colored green and M in cyan. N-glycosylated Asn are shown as blue spheres, palmitoylated cysteines as orange spheres and the disulfide-bond between Gp5 and M as red sticks.
Right part: Quality of the individual models displayed in rainbow colors ranging from red (high confidence) to blue (low confidence).

Figure S7

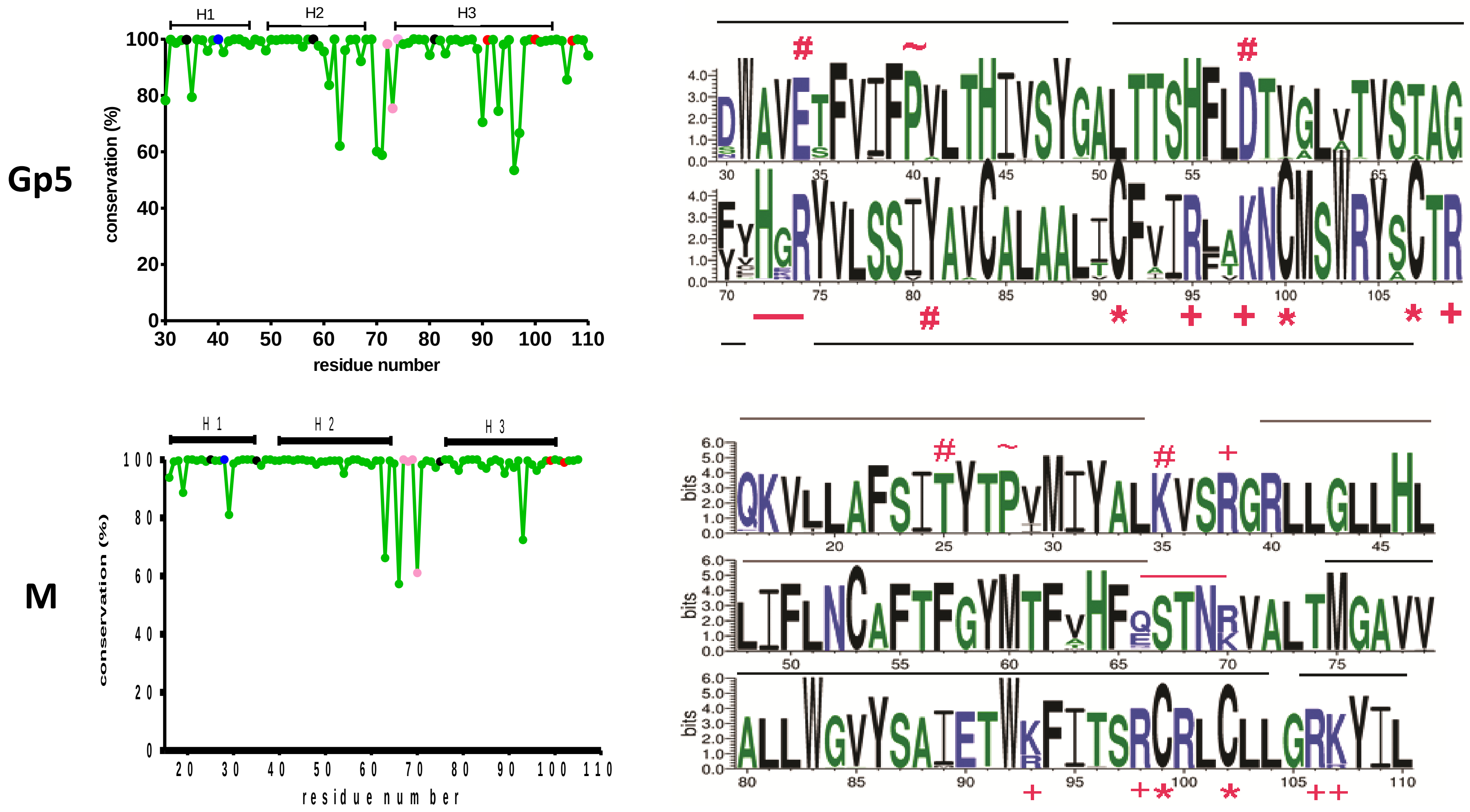

Figure S7: Conservation of amino acid residues in the transmembrane region of Gp5 and M of PRRSV-2 strains.
Left panel: The graphs show the percent conservation (y axis) of amino acids at each position (x axis) of a consensus sequence compiled from 7701 Gp5 sequences and 290 M sequences of PRRSV-2 present in the database. Red: acylated cysteine residues, blue: Proline in helix1, black: amino acids involved in hydrophilic contacts between Gp5 and M, pink: amino acids exposed at the molecule´s surface.
Right panel: Web loge representation of the same residues. #: amino acids involved in hydrophilic contacts between Gp5 and M, ~: Proline in helix 1, *: acylated cysteine residues, +: basic residues exposed to the open cavity. The three helices are marked with a black line and the amino acids exposed at the molecule´s surface by a red line.

Figure S8

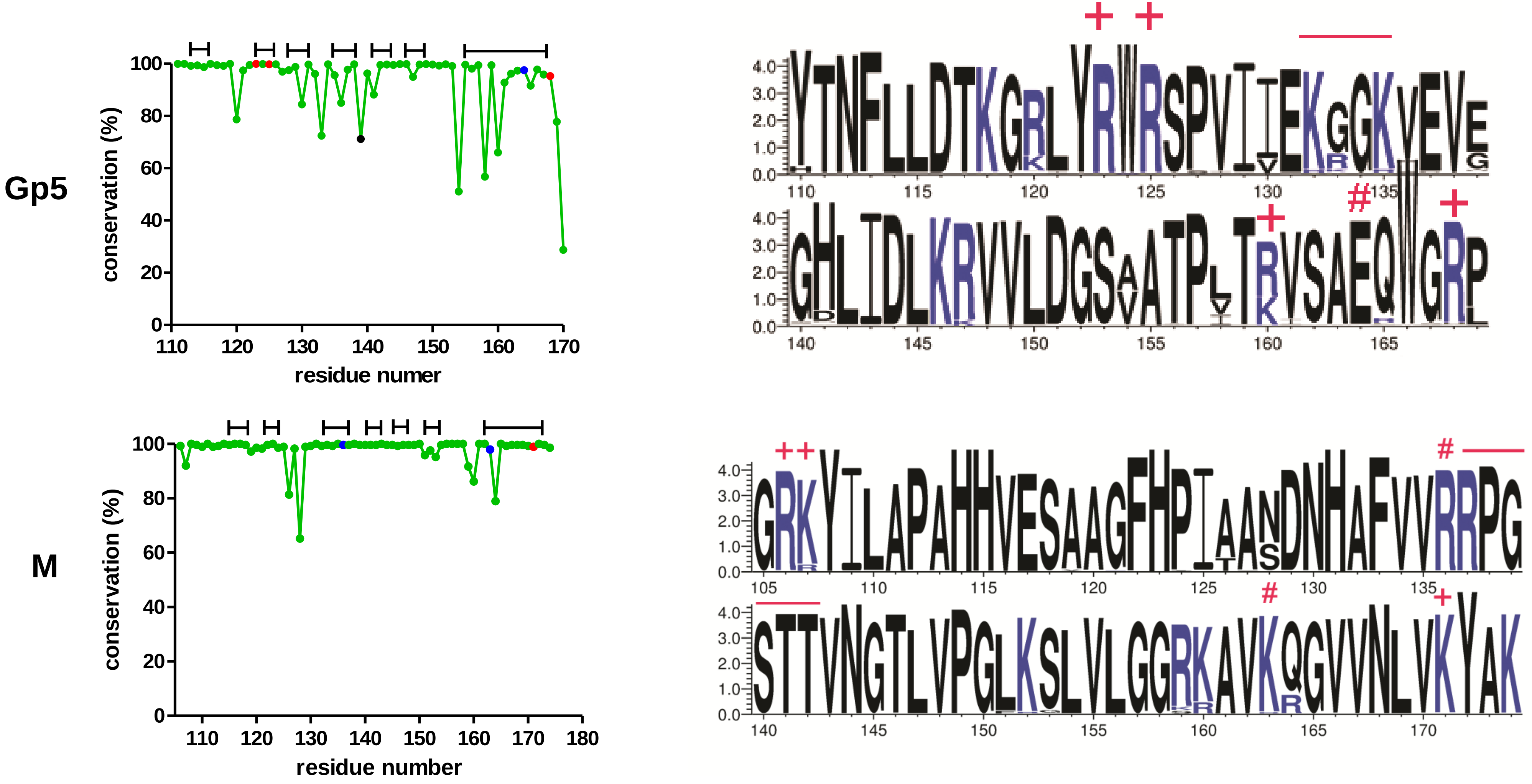

Figure S8: Conservation of amino acid residues in the endodomain of Gp5 and M of PRRSV-2 strains.
Left panel: The graphs show the percent conservation (y axis) of amino acids at each position (x axis) of a consensus sequence compiled from7701 Gp5 sequences and 290 M sequences of PRRSV-2 strains present in the database. Red: basic amino acids pointing into the basic open cavity. The location of the seven β-strands is indicated.
Right panel: Web loge representation of the same residues. #: amino acids involved in hydrophilic contacts between Gp5 and M, +: basic residues exposed to the open cavity. The residues marked with a red line are located between β3 and β4 and might interact with the viral nucleocapsid protein (see S27 Fig).

Figure S9

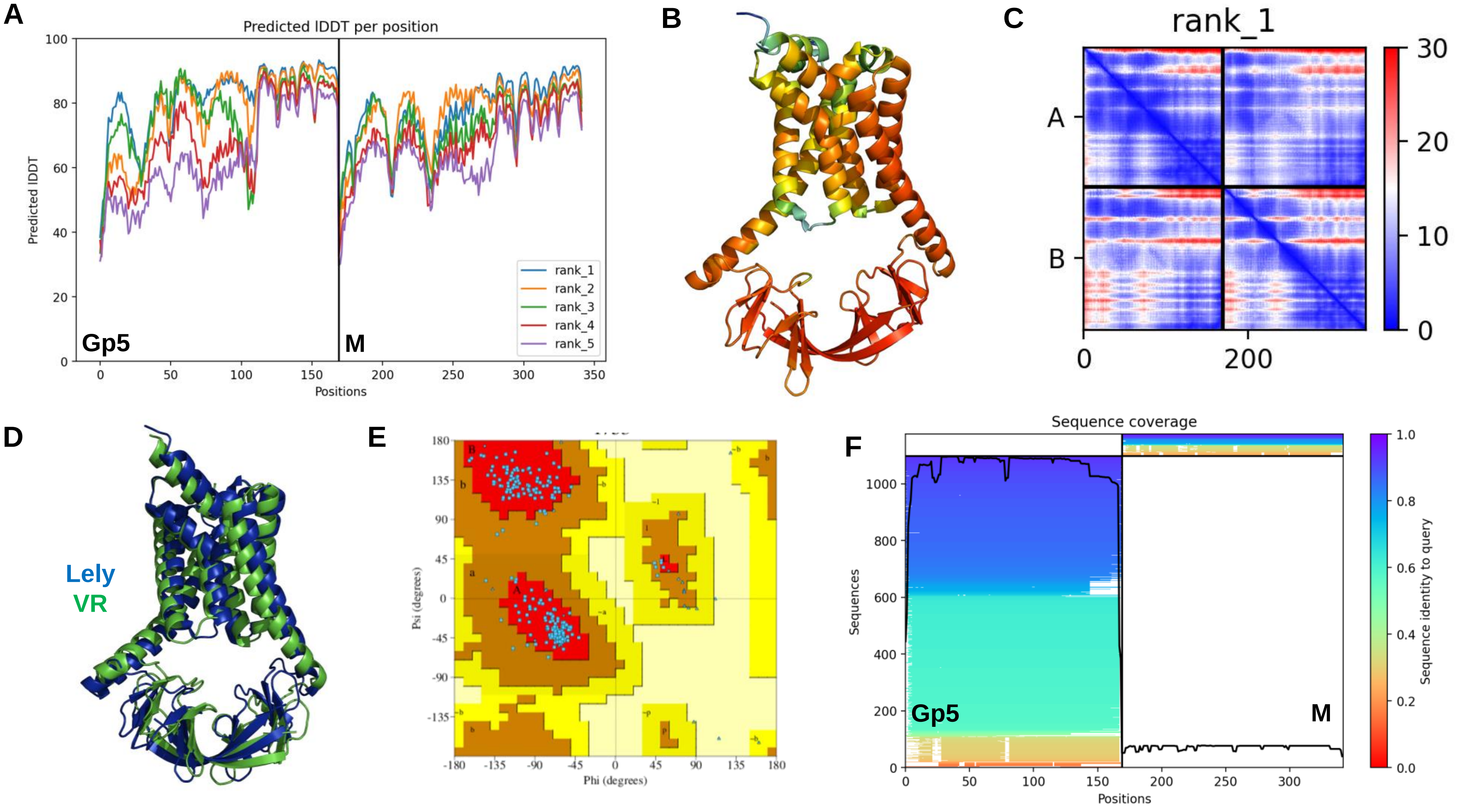

Figure S9: Reliability scores for the predicted structure of Gp5/M of PRSSV-1 Lelystad

A: Predicted local distance difference test (IDDT) score per position for the five models generated by alphafold2.

B: Cartoon model of the structure of Gp5/M showing the pIDDT per position in rainbow colors from red (high confidence) to blue (low confidence) **C:** Prediction aligned error (PAE) score for model 1. **D:** Gp5/M of Lelystad (blue) is superimposed with Gp5/M of VR 2332 (green). **E:** Ramachandran plot of the predicted structure of model 1. **F:** Multiple sequence alignment (MSA) file.

Figure S10

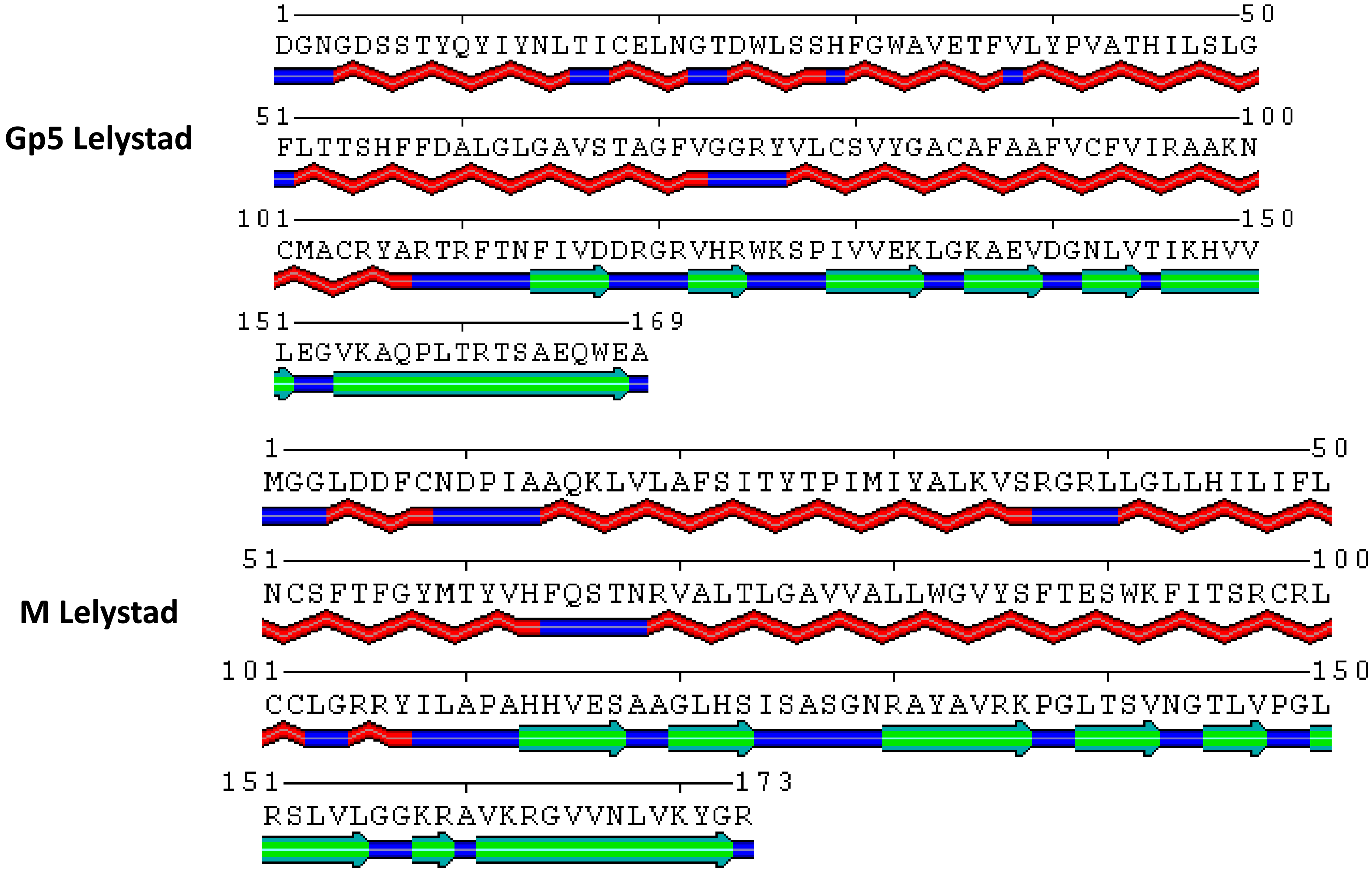

Figure S10: Assignment of Gp5 and M sequence of Lelystad to the predicted structural elements.
Red: α-helices, green: β-strands, blue: no structure, loops.

Figure S11

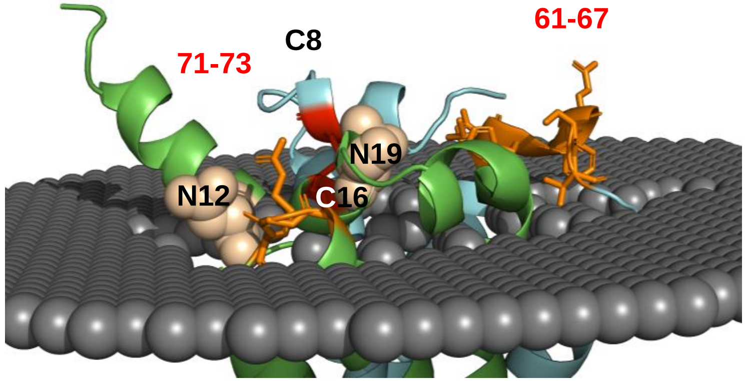

Figure S11: Integration of Gp5/M of Lelystad into a virtual lipid bilayer.
The border between the hydrophobic and hydrophilic part of the external bilayer is shown as grey spheres. Gp5 and M are shown as green and cyan cartoon, respectively. The N-glycosylation sites are shown as wheat spheres. Amino acids located in the loop between transmembrane helices 2 and 3 of both Gp5 and M and sticking out of the membrane are shown as orange sticks.

Figure S12

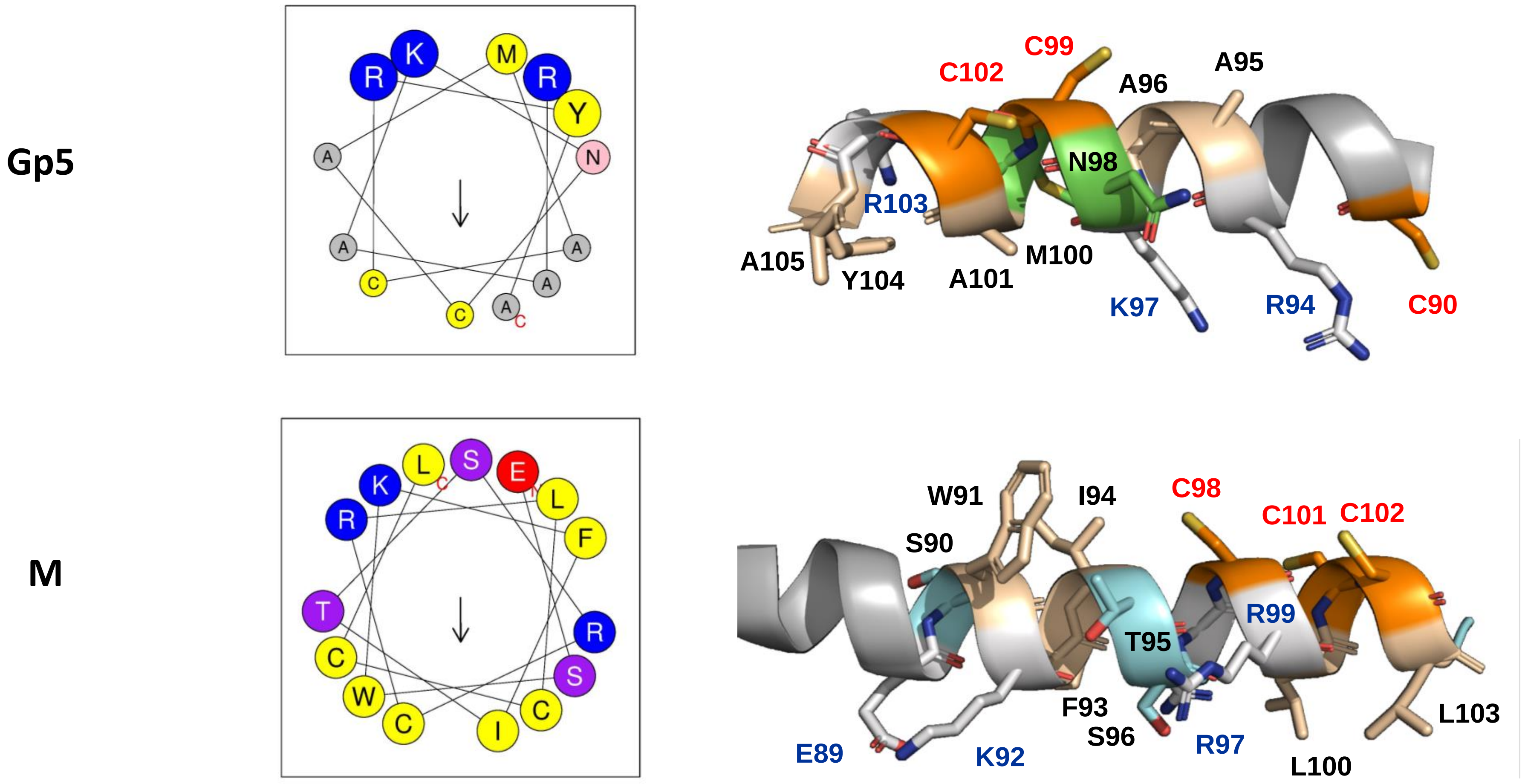

Figure S12: Structure of the amphiphilic helix at the C-terminal part of Gp5 and M from Lelystad
Left panel: Heliquest analysis of the C-terminal region of TM3 of Gp5 and M and **Right panel:** structure of the corresponding domain. Heliquest produces a helical wheel from the sequence with an arrow inside that points to the hydrophobic face. Its length corresponds to the hydrophobic moment. A hydrophobicity <H> of 0.247 and a hydrophobic moment <µH> of 0.342 was calculated for Gp5 whereas the corresponding values for M are <H> 0.693 and <µH>: 0.319.

Figure S13

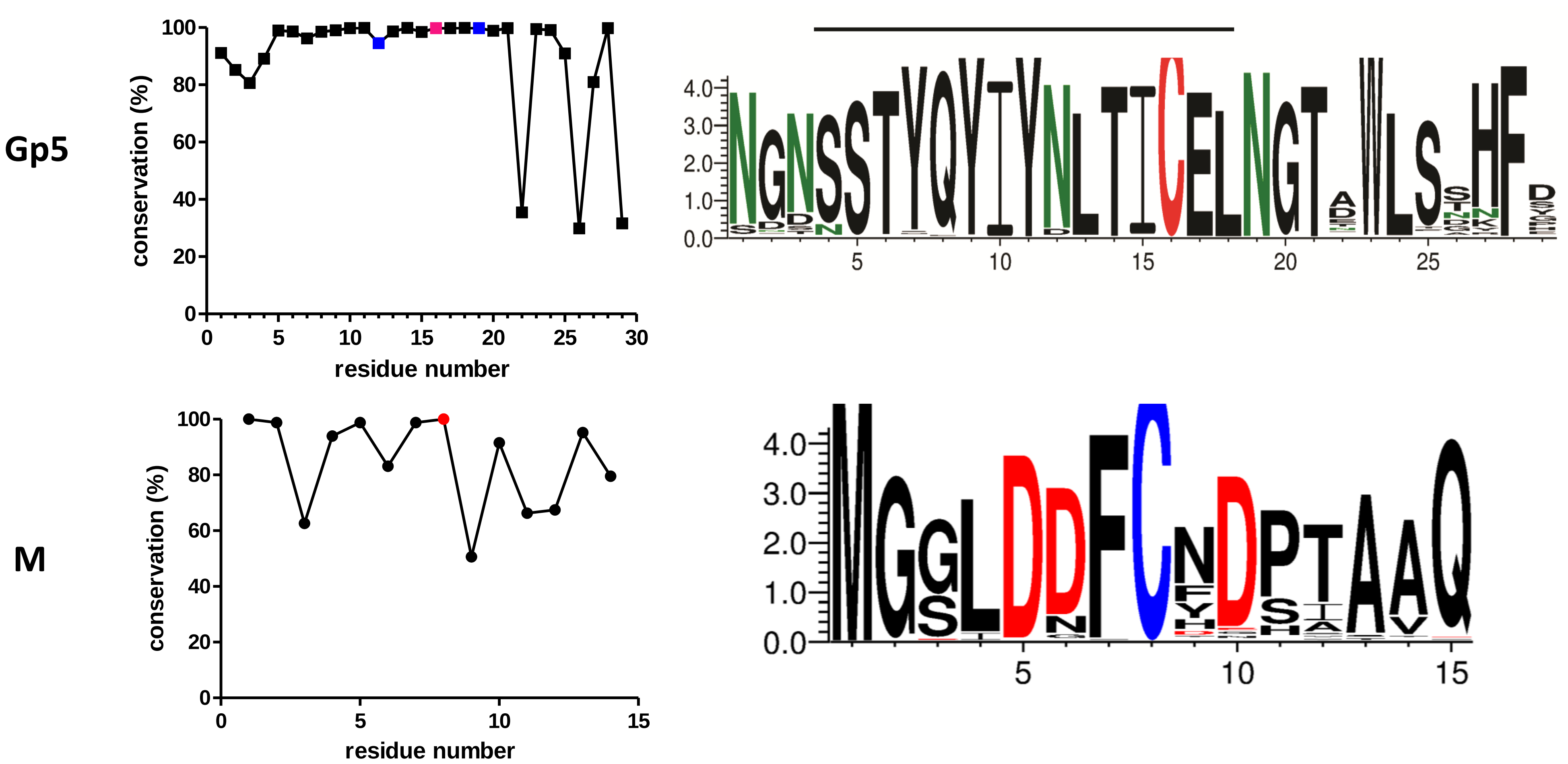

Figure S13: Conservation of amino acid residues in the ectodomain of Gp5 and M of PRRSV-1 strains.
Left panel: The graphs show the percent conservation (y axis) of amino acids at each position (x axis) of a consensus sequence compiled from 1077 Gp5 sequences and 83 M sequences of PRRSV-1 strains present in the database. Red: Cys forming the disulfide-bond between Gp5 and M. Blue: N-glycosylation sites.
Right panel: Web loge representation of the same residues. Green: Asn as putative N-glycosylation sites, Red or Blue: Cys forming the disulfide-bond between Gp5 and M, Red: acidic residues.

Figure S14

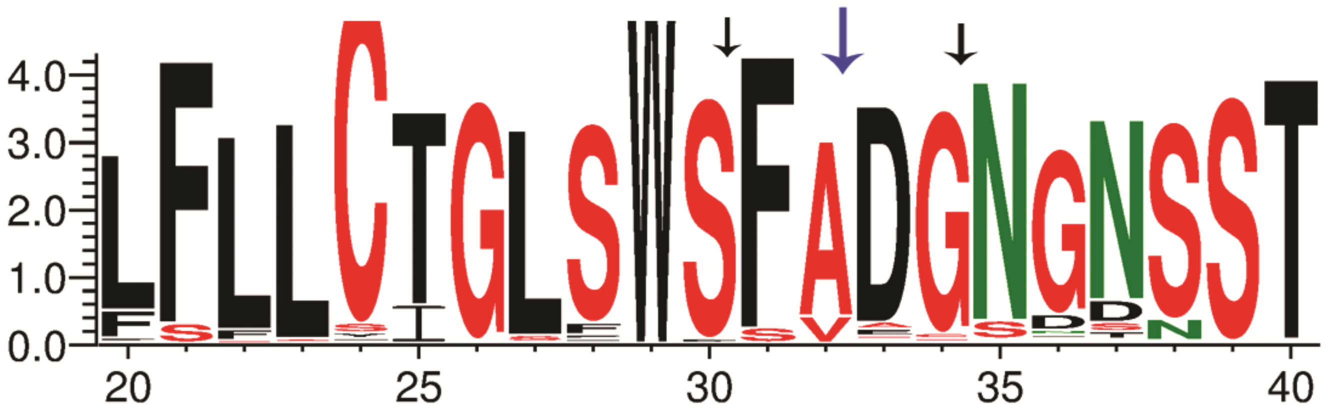

Figure S14: Web loge representation of amino acids 20 to 40 of full-length Gp5 of PRRSV-1 strains sequences including the signal peptide.
The blue, large arrow indicate the main cleavage site predicted by SignalP5 and the minor black arrows additional predicted cleavage sites. Small amino acids are shown in red and Asn residues as putative N-glycosylation sites are shown in green.

Figure S15

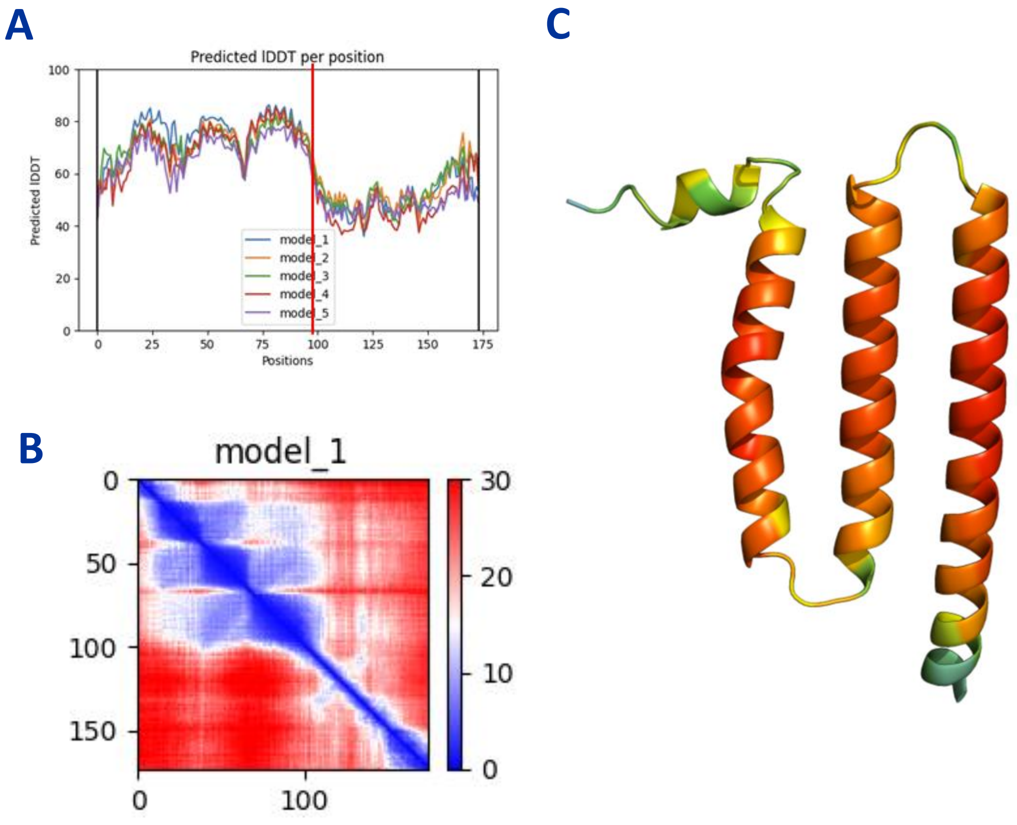

Figure S15: Predicted structure of monomeric M of PRSSV-2 VR 2332 and corresponding reliability scores
A: Predicted local distance difference test (lDDT) score per position for the five models generated by alphafold2. The red horizontal line separates the transmembrane region from the endodomain.
B: Prediction aligned error (PAE) score for model 1. **C:** Cartoon model of the structure of Gp5 and M showing the plDDT per position in rainbow colors from red (high confidence) to blue (low confidence). The endodomain was deleted from the structure, since the prediction was of low confidence and different folding were predicted for M of VR 2332 and Lelystad.

Figure S16

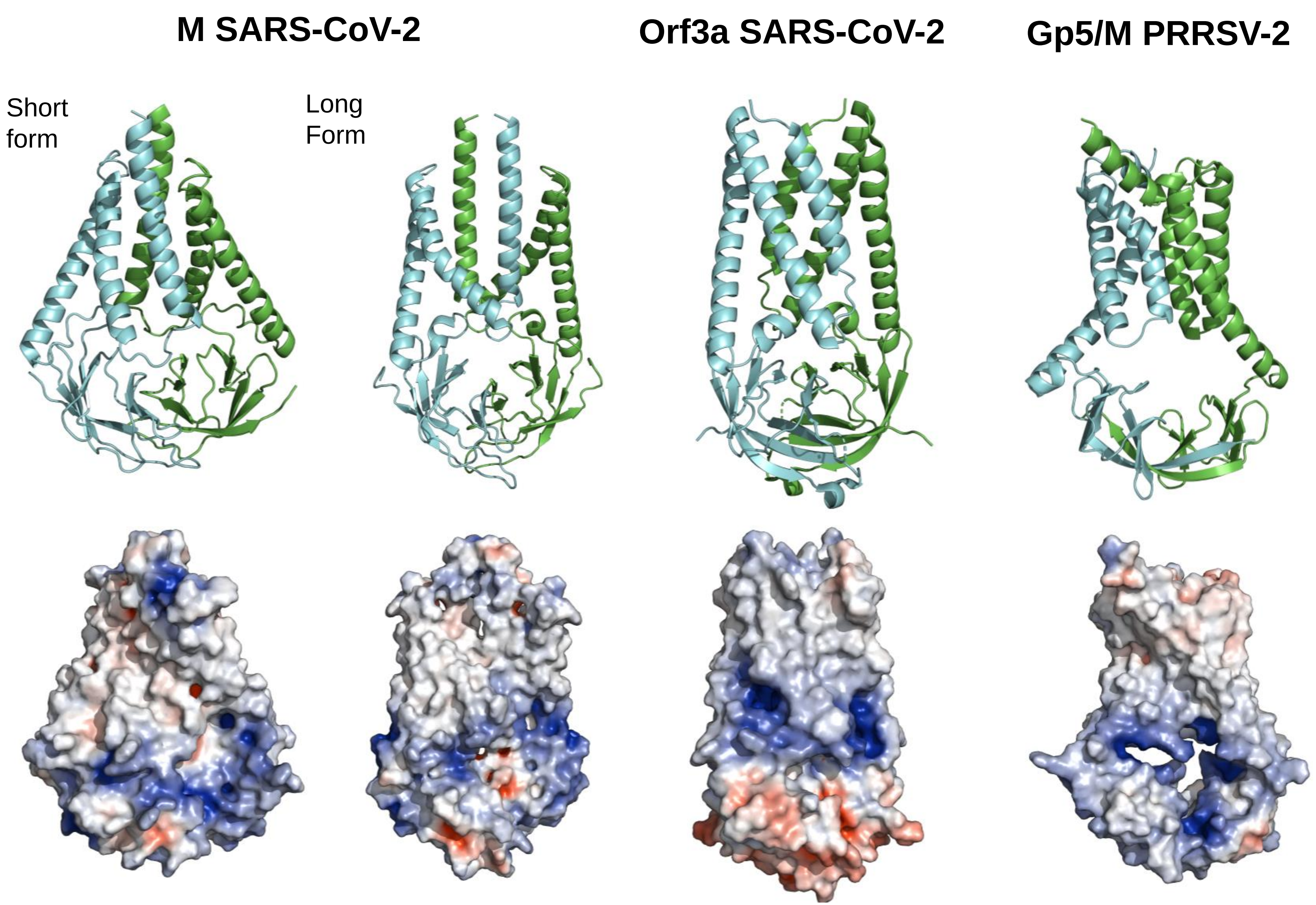

Figure S16: Experimentally determined structures of M and Orf3a of SARS-CoV-2.
Upper part: Cartoon model. Monomers are colored green or cyan. Gp5 is colored green and M in cyan in the predicted structure of Gp5/M of the PRRSV-2 reference strain VR 2332.
Lower part: Electrostatic surface potential of each dimer shows a positively charged region (blue) beneath the transmembrane part. Figures were created with Pymol from pdb-file 7VGS (M, short form), 7VGR (M. long form) and 6XDC (Orf3a).

Figure S17

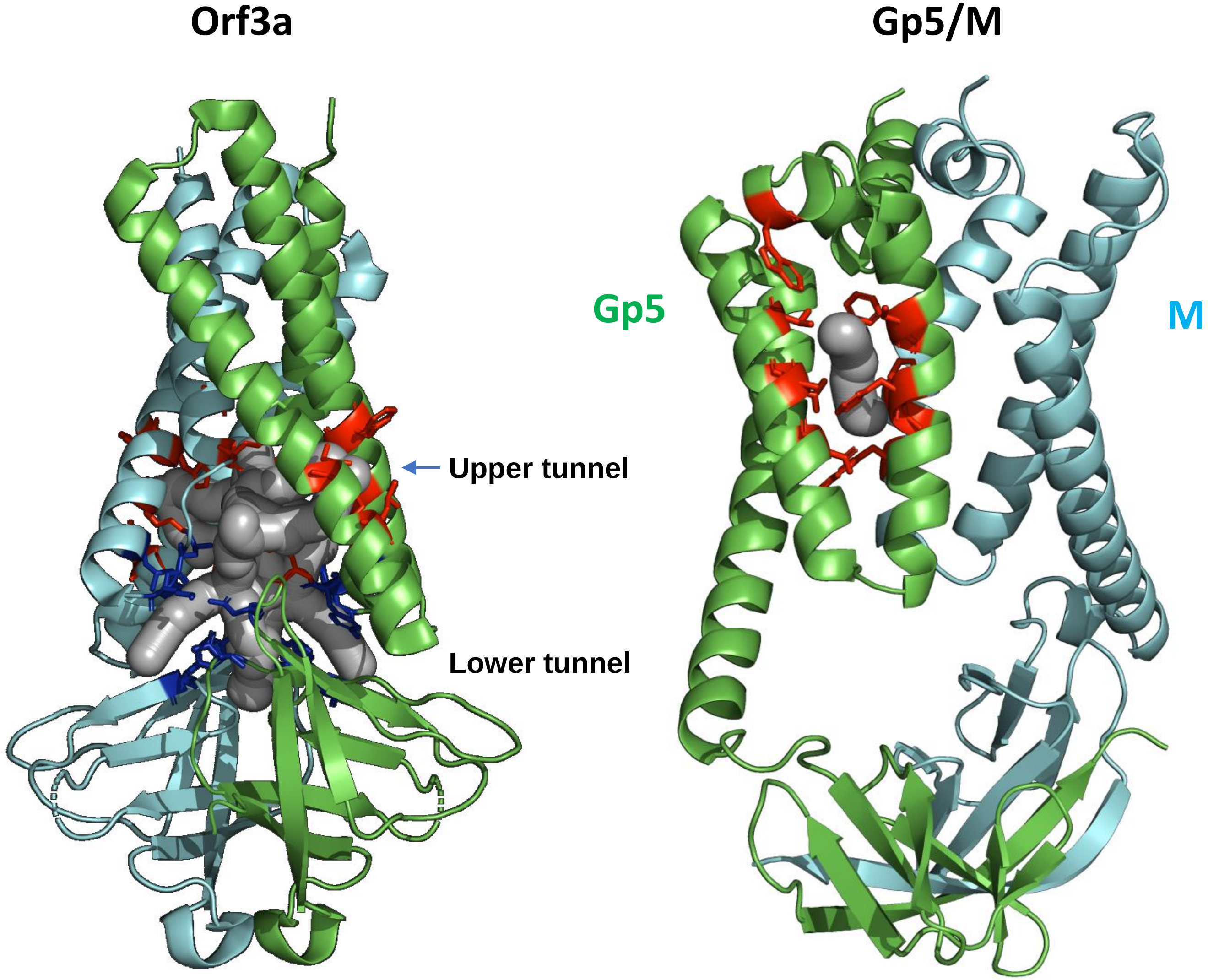

Figure S17: Cavities and tunnels in the transmembrane region of Orf3a and Gp5/M.
The tool Mole 2.5 was used to reveal cavities and tunnels within the structure of Orf3a and Gp5/M of VR 2332. The hydrophilic tunnels in Orf3a corresponds to tunnels described for the Cryo-EM structure, which are thought to conduct ions. Gp5/M of VR 2332 contains just one small cavity between TM1 and TM2 of Gp5, which is moreover lined with hydrophobic amino acids. Hence it is unlikely that Gp5/M function as an ion channel.

Figure S18

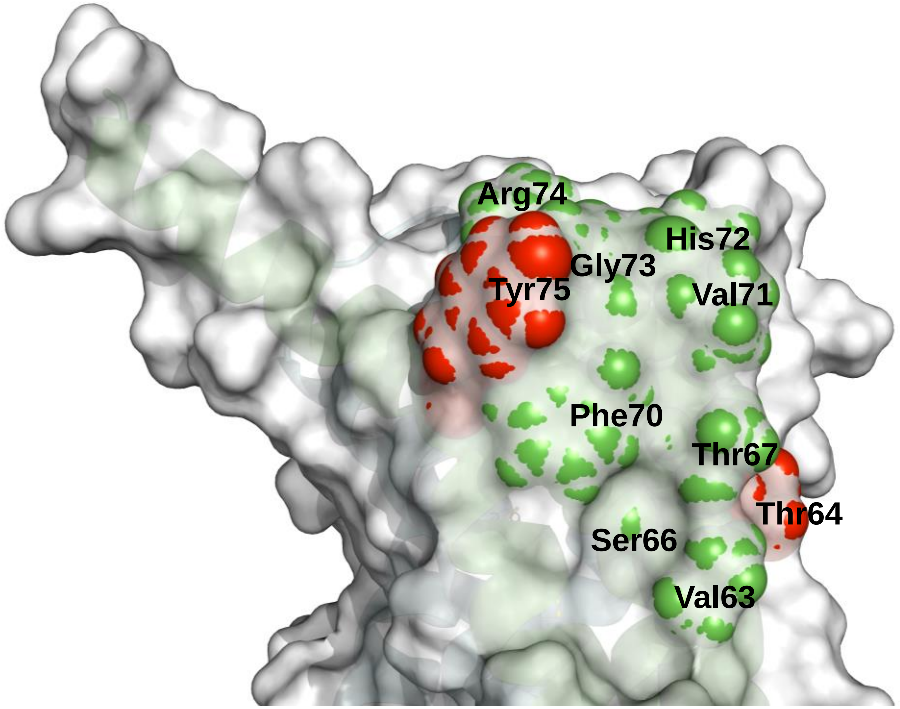

Figure S18: Surface representation of the outer part of the Gp5/M dimer of VR 2332 shows the location of the GADPH binding sites.
A: Amino acids that have been identified to interact with GADPH are highlighted as spheres. The amino acids highlighted in red have been shown to be positively selected after transmission of PRRSV from an unknown host to pigs.

Figure S19

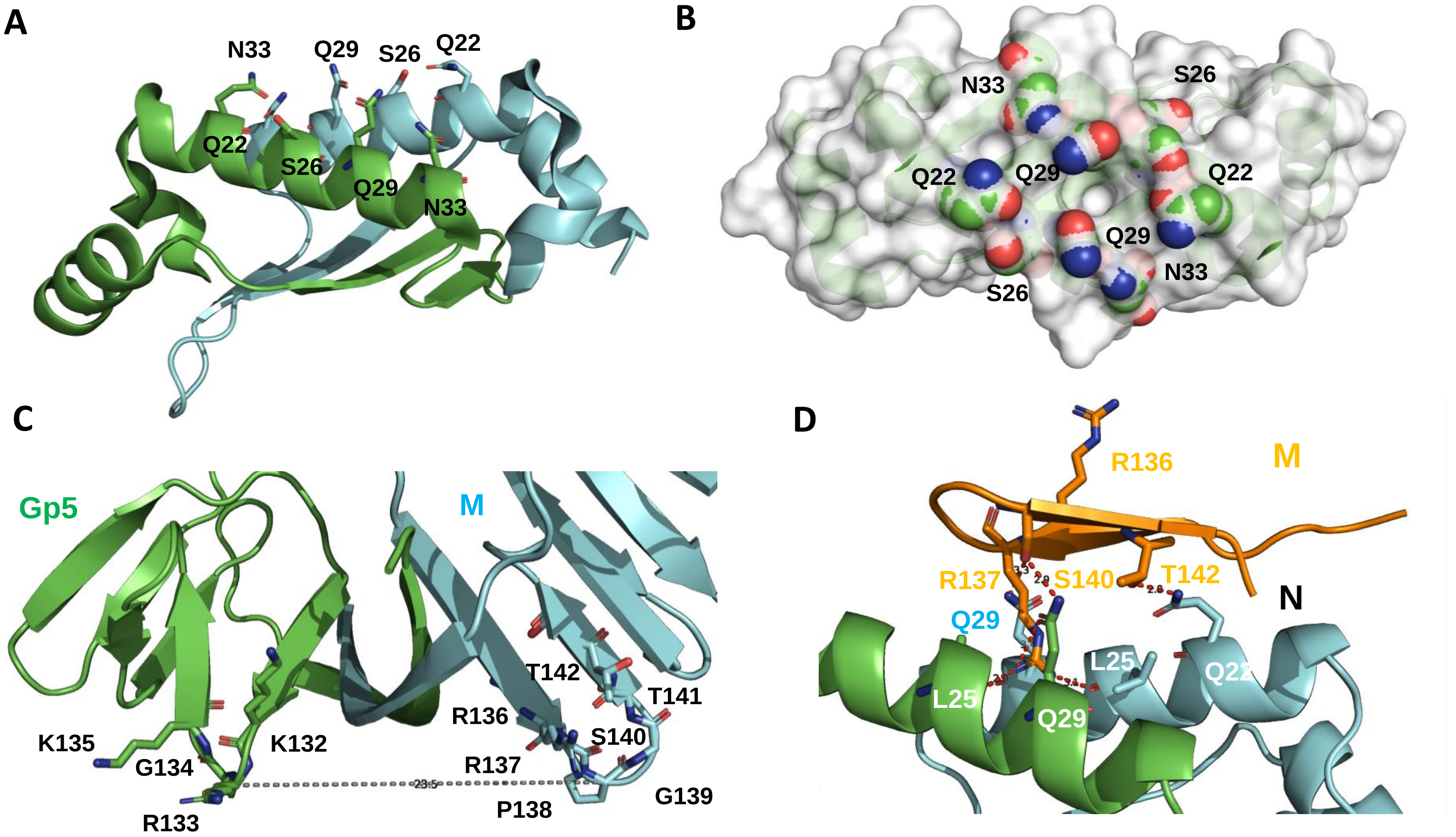

Figure S19: A putative binding site for the N-protein in the endodomain of M

A: Cartoon model of the C-terminal part of the nucleocapsid protein of PRRSV. One monomer is colored cyan, the other in green. **B:** Top view of a surface representation shows a groove with two indentations. Amino acids lining the groove are shown as sticks (A) or spheres (B). Figure created from pdb-file 1P65. **C:** Endodomain of the Gp5/M dimer showing basic residues in the loop between β3 and β4. **D:** Docking of the loop in M to N. Arg137 inserts into the large indentation and interacts with L25 in both helices of N. Ser140 interacts with Q29 of one and Thr142 with Q22 in the other helix of N.

Table S1

**Virus species** Predicted cleavage site	**Number of analyzed sequences and percent of total**	**The probability that the N-terminus is a signal peptide**	**The probability for the predicted cleavage site**	**Max/Min of the probabilities of the predicted cleavage sites**
**PRRSV-1**	**180**	**89,6** +-8,5	**54,0** +-12,0	**85,4-21,8**
Site 32-33	135 = 74,4	91,6 +-7,3	55,8 +-11,0	85,4-24,7
Site 34-35	30 =16,6	82,6 +-9,1	49,7 +-14,6	80,0-25,9
Site 30-31	14 = 7,7	86,0 +-9,7	46,4 +-11,2	59,8-21,8
Site 36-37	1	85,6	48,3	
**PRRSV-2**	**136**	**71,5** +-10,3	**47,0** +-14,2	**81,6 – 17.4**
Site 31-32	119 =87,5	71,6 +-10,4	49,1+-12,8	81,6 – 17.4
Site 29-30	7 = 5,1	74,3 +-9,2	35,9+-16,1	68,1 - 22,3
Site 26-27	6 = 4,4	68,3 +-7,5	34,3 +-16.7	65,9 -20,8
Site 24-25	1	72,1	28,0	
Site 27-28	1	73,5	19,5	
Site 28-29	1	53,0	17,5	
Site 33-34	1	84,0	23,9	

Table S1: Predicted signal peptide cleavage sites in Gp5 of PRRSV-1 and PRRSV-2 strains.
The indicated numbers of Gp5 sequences from PRRSV-1 and PRRSV-2 were analysed with SignalP5. The tool provides for each sequence a probability whether or not the N-terminus is an eukaryotic signal peptide, the exact cleavage site and the probability for this prediction. The mean of both probabilities including the standard deviation were calculated. Max and Min is the highest and lowest probability for the prediction of the cleavage site.

Table S2

N-terminal sequences of Gp5	Lineage	strain
MLGKCLTACCCSRLLFLWCIVPFYLAVLVNASNNNSSHIQLIYNLTLCELNGTDWLAQNF	3.4	JXA1-R
MLGKCLTACCCSRLLFLWCIVPFYLAVLVNASNNNSSHIQLIYNLTLCELNGTDWLAQKF	3.3	JXA1
MLGKCLTACCCSRLLFLWCIVPFYLAVLVNASNNNSSHIQLIYNLTLCELNGTDWLAQKF	3.5	HUB2
MLGKCLTACCCSRLLFLWCIVPFYLAVLVNASNNNSSHIQLIYNLTLCELNGTDWLAQKF	3.6	Bjsy06
MLGKCLTACCCSRLLFLWCIVPFYLAVLVNASNNNSSHIQLIYNLTLCELNGTDWLAQKF	3.7	WUH3
MLGKCLTAGYYSQLPFLWCIVPFCLVALANANNGSSSHLQLIYNLTICELNGTDWLNDHF	2.6	MN414
MLGKCLTAGYCSQLLFLWCIVPFCSIALVSASGNSSSYSQLIYNLTLCELNGTDWLASKF	2.2	HK2
MLGKCSTAGCCSQFLFLWCIVPFCSIALVSANGNSSSYSQLIYNLTLCELNGTDWLATNF	3.2	GM2
MLEKCLTAGCYSQLLSLWCIVPFCFAVLVNASNDSSSHLQLIYNLTLCELNGTDWLANKF	2.1	VR2332
MLGRCLTAGCCSRLLSLWCIVPFCFAALVNANSNSSSHLQLIYNLTLCELNGTDWLKDKF	1	JA142
MLGKCLTTGCCSRLLSLWCIVPFCFAVLVNANSNSSSHFQLIYNLTLCELNGTDWLANKF	3.1	CH-1a
MLGKCSTAGCCSPLLFLWCIVPSCLVALVNASKNNSSHLQSIYNLTICELNGTDWLNKKF	2.5	NADC31
MLGKCLTAGYCSQLPFLWCIVPFCLAALVNADSNSSSHLQLIYNLTICELNGTDWLNNHF	2.4	MN184C
MLGKCLTAGYCSRLPFLWCIVPFCFAALVNASGNSSSHLQLIYNLTICELNGTDWLEARF	2.3	CA-2
MLGKCLTAGYCSQLPFLWCIVPFCFAALVNANSNSSSHLQLIYNLTICELNGTDWLNERF	2.7	NADC31
** :* *: * : ****** .*..*. ..**: * *****:********* .*		

N-terminal sequences of Gp5	Accession No	Probability
MLGKCLTARCCSRLLFLWCIVPFCLTVLVNADGNSSSHIQLIYNLT	QDF82135	0,82
MLGRCLTAGCCSRLPSLWCIVPFCFAALVNAGNDNSSHLQLIYNLT	ACG52080	0,77
MSEKCLTACYCLPSLFLWCIVPFCSIALASDGGNSSSYSQLIYNLT	QDF82151	0,68
MLGKCLTAGCCSRLPFLWCIVPFCFAVIDNVSGNSSSHFQLIYNLT	AAT01122	0,66

Table S2: N-terminal Gp5 sequences and predicted signal peptide cleavage sites

Upper panel: N-terminal Gp5 sequences from the representative members of the three clades of PRRSV-2 strains and their sublineages (Guo et al., Viruses 2021, 13, 2469). The main cleavage site predicted by SignalP5 is highlighted in green and minor cleavage sites predicted for some Gp5 proteins are highlighted in blue. **Lower panel:** N-terminal Gp5 sequences predicted with the highest probability to be cleaved between residues 31 and 32, 29 and 30 and 26 and 27 (highlighted in green).

Small amino acids which allow peptide cleavage if present at the -1 and -3 position relative to the cleavage site are in red. Large residues which prevent cleavage at a certain site are in blue. Putative N-glycosylation sites around the signal peptide cleavage site are highlighted in grey.

Table S3

	Gp5 VR	Gp5 Lely	M VR	M Lely	Orf3a SARS	M SARS
**Gp5 VR**	X	62	10	12	15	15
**Gp5 Lely**	61 / 72	X	11	11	16	15
**M VR**	18 / 33	18 / 28	X	78	13	18
**M Lely**	19 / 30	18 / 31	79 / 91	X	14	20
**Orf3a SARS**	4 / 6	10 / 17	15 / 21	11 / 21	X	12
**M SARS**	15 / 25	20 / 29	16 / 28	15 / 30	14 / 23	X

Table S3: Amino acid sequence conservation between Gp5 and M of Arteriviruses and Orf3a and M of SARS-CoV-2. The numbers above the diagonal where generated by a multiple sequence alignment of all amino acid sequences using Clustal Q https://www.ebi.ac.uk/Tools/msa/clustalo/ and are the calculated identity matrix.
The numbers below the diagonal were generated by a pairwise sequence alignment using needle https://www.ebi.ac.uk/Tools/psa/emboss_needle/ and are the calculated percentage of identical and similar amino acids, respectively for each pairwise alignment. The predicted signal peptides of Gp5 were removed from the sequence prior to both analysis.
